# Quantitative proteomic view associated with resistance to clinically important antibiotics in Gram-positive bacteria: a systematic review

**DOI:** 10.3389/fmicb.2015.00828

**Published:** 2015-08-11

**Authors:** Chang-Ro Lee, Jung Hun Lee, Kwang Seung Park, Byeong Chul Jeong, Sang Hee Lee

**Affiliations:** National Leading Research Laboratory of Drug Resistance Proteomics, Department of Biological Sciences, Myongji UniversityYongin, South Korea

**Keywords:** quantitative proteomics, methicillin resistance, vancomycin resistance, linezolid resistance, daptomycin resistance

## Abstract

The increase of methicillin-resistant *Staphylococcus aureus* (MRSA) and vancomycin-resistant *Enterococcus* (VRE) poses a worldwide and serious health threat. Although new antibiotics, such as daptomycin and linezolid, have been developed for the treatment of infections of Gram-positive pathogens, the emergence of daptomycin-resistant and linezolid-resistant strains during therapy has now increased clinical treatment failures. In the past few years, studies using quantitative proteomic methods have provided a considerable progress in understanding antibiotic resistance mechanisms. In this review, to understand the resistance mechanisms to four clinically important antibiotics (methicillin, vancomycin, linezolid, and daptomycin) used in the treatment of Gram-positive pathogens, we summarize recent advances in studies on resistance mechanisms using quantitative proteomic methods, and also examine proteins playing an important role in the bacterial mechanisms of resistance to the four antibiotics. Proteomic researches can identify proteins whose expression levels are changed in the resistance mechanism to only one antibiotic, such as LiaH in daptomycin resistance and PrsA in vancomycin resistance, and many proteins simultaneously involved in resistance mechanisms to various antibiotics. Most of resistance-related proteins, which are simultaneously associated with resistance mechanisms to several antibiotics, play important roles in regulating bacterial envelope biogenesis, or compensating for the fitness cost of antibiotic resistance. Therefore, proteomic data confirm that antibiotic resistance requires the fitness cost and the bacterial envelope is an important factor in antibiotic resistance.

## Introduction

Antibiotic resistance has posed a serious threat to the worldwide public health in the past two decades. The gradual increase in resistance rates of several important pathogens, including methicillin-resistant *Staphylococcus aureus* (MRSA), vancomycin-resistant *Enterococcus* (VRE), multidrug-resistant (MDR) *Pseudomonas aeruginosa*, imipenem-resistant *Acinetobacter baumannii*, and third-generation cephalosporin-resistant *Escherichia coli* and *Klebsiella pneumonia*, has become an increasingly severe problem in many hospitals worldwide (Lee et al., 2013). However, the decline in novel antibiotics that are introduced in the market weakens the hope of overcoming this threat by the development of new antibiotics. Most of the antibiotic classes used in hospitals today were discovered during the period 1930–1960. Only two new systemic classes of antibiotics that were developed during the past 30 years were linezolid and daptomycin, which are used only in the treatment of Gram-positive pathogens (Lee et al., 2013). Because many Gram-positive pathogens increasingly develop resistance against currently available antibiotics such as methicillin and vancomycin, these new antibiotics have become valuable for the treatment of various infections of methicillin- or vancomycin-resistant *S. aureus* and *Streptococcus pneumonia* (Ament et al., 2002; Mendes et al., 2014). However, the emergence of daptomycin-resistant or linezolid-resistant strains has recently been described in some Gram-positive pathogens (Fischer et al., 2011; Mendes et al., 2014). In this review, we summarize resistance mechanisms to four clinically important antibiotics (methicillin, vancomycin, linezolid, and daptomycin) used in the treatment of Gram-positive pathogens, and highlights recent important studies using comparative proteomic tools to understand resistance mechanisms of these antibiotics in more detail.

## Action and resistance mechanisms of methicillin, vancomycin, linezolid, and daptomycin resistance

### Methicillin

Methicillin is a narrow-spectrum β-lactam antibiotic of the penicillin class. Like other β-lactam antibiotics, methicillin prevents the synthesis of bacterial cell walls by inhibiting peptidic cross-linkage between the linear peptidoglycan polymer chains, which provides rigidity to the cell wall of Gram-positive bacteria (Chambers, 1997) (Table 1). Methicillin and other β-lactam antibiotics are structural analogs of D-Ala-D-Ala, which is the terminus of a short amino acid chain attached in *N*-acetylmuramic acids; so, they interact with and irreversibly inhibit the transpeptidase enzyme [also called penicillin-binding protein (PBP)] that crosslinks the linear peptidoglycan polymer chains (Lee et al., 2012). This process leads to loss of osmotic integrity and makes the bacterial cells susceptible to lysis. Although most β-lactam antibiotics are inhibited by bacterial enzymes that hydrolyze the β-lactam ring (named β-lactamases), due to a modification of the original penicillin structure methicillin is resistant to β-lactamases (Lee et al., 2012). Therefore, since the late 1950s when methicillin was first introduced in markets, this antibiotic has been used to treat infections caused by *Staphylococcus* pathogens such as *Staphylococcus aureus*, most of which produces β-lactamase (Newsom, 2004).

**Table 1:** **Modes of action of four clinically important antibiotics (methicillin, vancomycin, linezolid, and daptomycin) and resistance mechanisms to these antibiotics**.

**Antibiotics**	**Target**	**Mechanism of action**	**Resistance mechanisms found by non-proteomic approaches**
Methicillin	Transpeptidase enzyme [penicillin-binding protein (PBP)]	Inhibition of peptidoglycan biosynthesis	Expression of penicillin-binding protein 2a (MecA), efflux pump
Vancomycin	D-Ala-D-Ala dipeptide terminus of the nascent peptidoglycan	Inhibition of peptidoglycan biosynthesis	Alteration of the D-Ala-D-Ala dipeptide
Linezolid	23S rRNA	Inhibition of translation	Alteration of 23S rRNA
Daptomycin	Cell membrane	The formation of holes that leak intracellular ions	Remained to be elucidated

Today, methicillin is not as effective against these organisms due to resistance (Cordwell et al., 2002; Newsom, 2004). Although the resistance phenotype of methicillin is influenced by numerous factors, including *mecA, glmM, fmtAB, murE, llm*, β-lactamase (*bla*) regulatory elements, and *fem* factors (Chambers, 1997; Cordwell et al., 2002; Hao et al., 2012), one major reason for methicillin resistance is the expression of the *mecA* gene, encoding penicillin-binding protein 2a (PBP 2a) that is not inhibited by classical β-lactam antibiotics including methicillin (Katayama et al., 2004) (Table 1). PBP 2a works in a similar manner to other PBPs, but it is bound by β-lactams with very low affinity (Katayama et al., 2004). Expression of PBP 2a confers resistance to all β-lactams. A variety of factors such as MecI and MecR1 controlled the *mecA* expression (Chambers, 1997). Resistance to methicillin exhibited by strains lacking the *mecA* gene is associated with modifications in native PBPs, β-lactamase hyperproduction, or possibly a methicillinase (Chambers, 1997). In pathogenesis, it has been reported that some virulence factors (Panton-Valentine leukocidin, phenol-soluble modulin, arginine catabolic mobile element, and other toxin elements) and two-component regulation systems (*agr, saeRS*, and *vraRS*) involved in pathogenesis can enhance the fitness of methicillin-resistant pathogens (Hao et al., 2012).

### Vancomycin

Vancomycin made by the soil bacterium *Amycolatopsis orientalis* is a member of the glycopeptide antibiotic class and has an important role in the treatment of serious infections caused by Gram-positive bacteria such as *Staphylococcus* and *Streptococcus* (Woodford, 1998). It is a complex compound consisting of a branched tricyclic glycosylated peptide and is a rare example of a halo-organic natural compound containing two covalently bonded chlorine atoms (Levine, 2006). Vancomycin inhibits the peptidoglycan synthesis by binding at the D-Ala-D-Ala dipeptide terminus of the nascent peptidoglycan in Gram-positive bacteria (Healy et al., 2000; Levine, 2006). This binding of vancomycin to the D-Ala-D-Ala prevents the peptidic cross-linking between the linear peptidoglycan polymer chains by inhibiting the proper interaction with the transpeptidase enzyme (Healy et al., 2000) (Table 1).

Most Gram-negative bacteria are intrinsically resistant to vancomycin because it cannot penetrate the outer membrane of Gram-negative bacteria. In Gram-positive bacteria, one mechanism of resistance to vancomycin is the alteration of the terminal amino acid residues (D-Ala-D-Ala), to which vancomycin binds (Table 1). The D-Ala-D-Ala dipeptide terminus of the nascent peptidoglycan is replaced by D-Ala-D-Lac or D-Ala-D-Ser. The D-Ala-D-Lac variation results in a 1000-fold decrease in the affinity between vancomycin and the peptide, and the D-Ala-D-Ser variation causes a 6-fold loss of affinity, most likely due to steric hindrance (Courvalin, 2005). These alterations of the D-Ala-D-Ala dipeptide terminus require the coordinate action of several enzymes encoded by the *van* genes. Alternative ligases catalyze the formation of the D-Ala-D-Lac peptide (VanA, B, and D type enzymes) or D-Ala-D-Ser peptide (VanC, E, and G type enzymes) in peptidoglycan synthesis. VanH protein (α-keto acid reductase) reduces pyruvate to D-Lac, and the D,D-dipeptidase VanX selectively removes the D-Ala-D-Ala produced by the native ligase to enhance the incorporation of the D-Ala-D-Lac or D-Ala-D-Ser into the peptidoglycan precursor. VanR and VanS constitute a two-component regulatory system that activates the transcription of the *van* gene cluster (Marcone et al., 2010).

### Linezolid

Linezolid is a first synthetic oxazolidinone antibiotic used to treat infections caused by VRE and MRSA. Although the mechanism of action of linezolid is not fully understood, it seems to bind to the 50S subunit of the bacterial ribosome through interaction with the central loop of the 23S rRNA and block the formation of protein synthesis initiation complexes (Swaney et al., 1998; Ament et al., 2002) (Table 1). Because linezolid binds to the 23S portion of the 50S subunit different from the binding sites of other ribosome-binding antibiotics such as chloramphenicol, cross-resistance between linezolid and other protein synthesis inhibitors is highly rare (Herrmann et al., 2008). The crystal structures of linezolid bound to the 50S subunit in 2008 showed that linezolid binds to the A site of the 50S ribosomal subunit and induces a conformational change perturbing the correct positioning of tRNAs on the ribosome (Ippolito et al., 2008; Wilson et al., 2008).

Most Gram-negative bacteria have an intrinsic resistance to linezolid due to the high activity of efflux pumps, which actively pump linezolid out of the cell (Schumacher et al., 2007). In Gram-positive bacteria, the acquired resistance to linezolid was first reported in 1999 in multidrug-resistant *Enterococcus faecium* (Mendes et al., 2014). High-resolution structures of linezolid with the 50S ribosomal subunit showed that it binds to a deep cleft that is surrounded by the central loop of domain V of 23S rRNA (Long and Vester, 2012). Therefore, the most common resistance mechanism of Gram-positive bacteria to linezolid was a point mutation known as G2576T, in which the G2576 position of 23S ribosomal RNA is converted to thymine (Mendes et al., 2014). In addition to mutations in 23S rRNA, other mechanisms have been identified in Gram-positive bacteria, including a six base pair deletion in the ribosomal protein L4, mutations in the ribosomal protein L3, mutations in an RNA methyltransferase (encoded by the *cfr* gene) that methylates G2445 of the 23S rRNA, and mutations causing increased expression of ABC transporter genes (*patA* and *patB*).

### Daptomycin

Daptomycin is a lipopeptide antibiotic consisting of a lipid molecule conjugated with anionic peptide and is a natural compound found in the soil bacterium *Streptomyces roseosporus* (Miao et al., 2005). Daptomycin absolutely requires Ca^2+^ for activity, making this agent a cationic antimicrobial peptide functionally (Baltz, 2009). The poorly calcium-decorated form of daptomycin is 10 times less active microbiologically than the heavily calcium-decorated form (Baltz, 2009). The calcium-bound daptomycin interacts with phosphatidylglycerol in the bacterial membrane and inserts into the cell membrane, leading to the formation of holes that leak intracellular ions (Pogliano et al., 2012). A loss of membrane potential causes inhibition of protein, DNA, and RNA synthesis, which results in bacterial cell death (Pogliano et al., 2012). Because of a distinct mechanism of action of daptomycin, it is used in the treatment of life-threatening infections caused by multiple drug-resistant Gram-positive bacteria (Baltz, 2009). Because vancomycin and daptomycin have molecular weight (MWs) of more than 1000 Da (vancomycin of 1449 Da and daptomycin of 1620 Da), they cannot penetrate the outer membrane of Gram-negative bacteria (Lee et al., 2013). Therefore, two antibiotics are used to control infections caused by Gram-positive bacteria.

Although daptomycin was clinically introduced in 2003, clinical treatment failures by the emergence of daptomycin-resistant strains during therapy have now been described (Hobbs et al., 2008; Fischer et al., 2011). Up to now, specific genetic determinant of the daptomycin-resistant strain remained to be elucidated, despite the finding of several phenotypic and genetic determinants (altered phospholipid synthesis, thickened cell walls, alteration of cell membrane fluidity, and the acquisition of mutations within the *mprF* or *yycG* gene) (Mishra et al., 2009; Fischer et al., 2011). The *mprF* gene encodes a dual functional enzyme that catalyzes the coupling of lysine to phosphatidylglycerol (PG) and transfers the lysyl-PG (LPG) to the outer leaflet of the membrane. The LPG is less acidic than PG, and membranes lacking LPG are more acidic than those containing PG and LPG (Baltz, 2009). Daptomycin-resistant strains with *mprF* mutations have membranes with increased levels of LPG (Jones et al., 2008). Therefore, the increased positive charge caused by increased LPG in the *mprF* mutant (gain-of-function) reduces the binding of Ca^2+^-bound daptomycin to bacterial membranes by a less favorable electrostatic interaction. YycG is a membrane spanning sensor histidine kinase of a two-component signal transduction system that partners with the YycF response regulator. YycFG functions as a master regulatory system for cell wall metabolism and biofilm formation and is the only two-component system required for viability in many Gram-positive bacteria (Winkler and Hoch, 2008; Baltz, 2009).

## Comparative proteomic analyses of methicillin, vancomycin, linezolid, and daptomycin resistance

**Figure 1:**
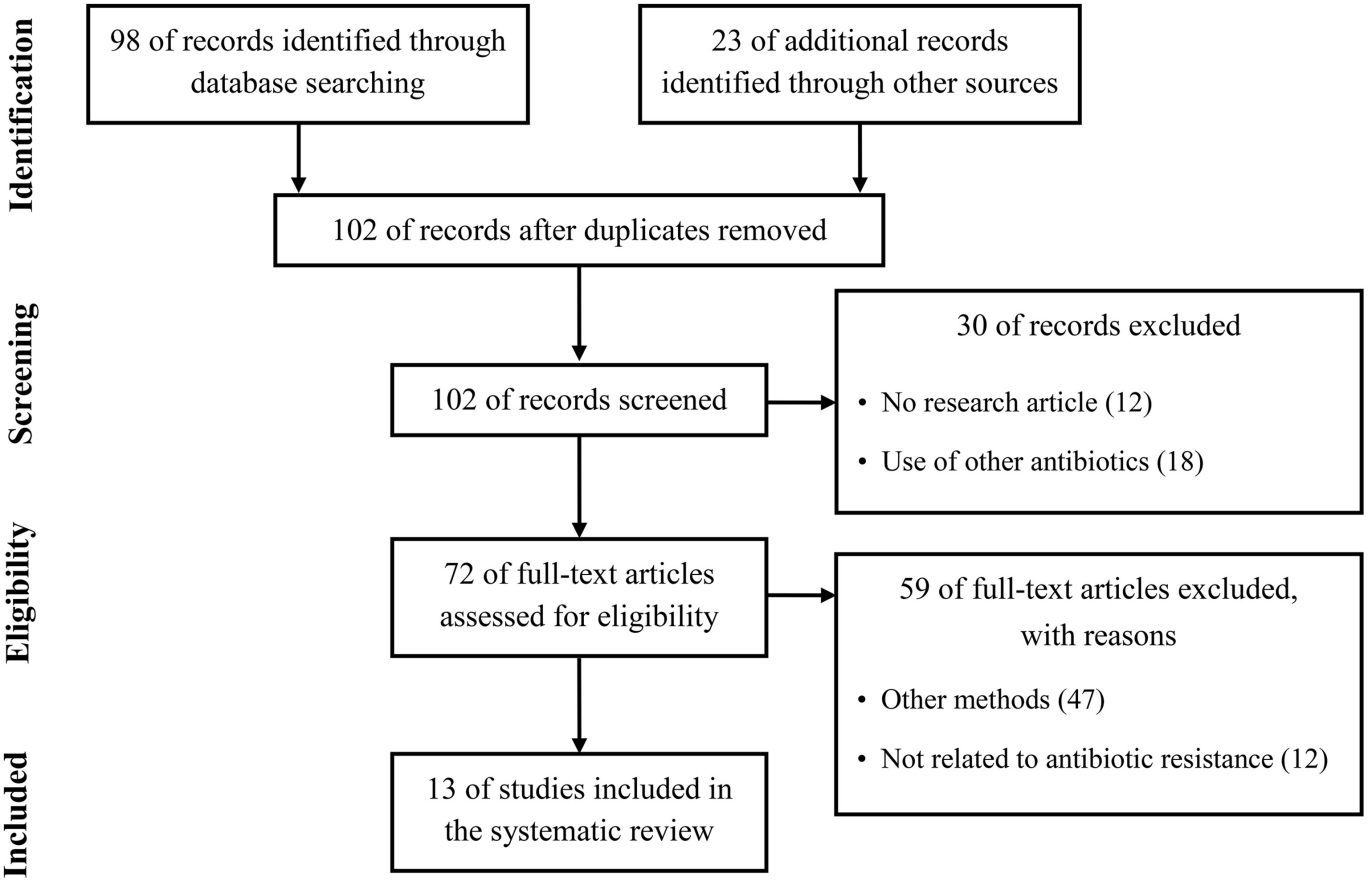
**Literature selection process (PRISMA flow diagram)**.

Quantitative proteomics have been considerably improved during the past decade and have been employed for investigation of the differences in whole protein expression dynamics of cells grown under a variety of growth conditions or stress conditions such as antibiotics (Radhouani et al., 2012). Therefore, by studies using quantitative proteomic approaches in the past few years, a considerable progress has recently been made in the study of antibiotic resistance mechanism. To summarize recent updates to understand the resistance mechanism to four clinically important antibiotics used in the treatment of Gram-positive pathogens, we used the Preferred Reporting Items for Systematic Review and Meta-Analysis (PRISMA) in our review (Figure 1) (Moher et al., 2009). We conducted a systematic literature search in the following databases: Medline via PubMed and Embase. We used keywords as search terms. We combined terms for selected indications (methicillin, vancomycin, linezolid, daptomycin, and proteomics). The literature search included all studies published in English between 2000 and 2015. We identified 13 proteomics studies comparing proteomic profiles in antibiotic-resistant and antibiotic-sensitive strains or exploring proteomic profiles in cells treated with or without antibiotics.

### Methicillin

Two studies exploring proteomic profiles of methicillin-susceptible *S. aureus* (MSSA) and methicillin-resistant *S. aureus* (MRSA) in the absence of methicillin were reported (Cordwell et al., 2002; Enany et al., 2014). Cordwell et al. compared the protein profiles between *S. aureus* strains COL (methicillin-resistant) and 8325 (methicillin-susceptible) in the absence of methicillin (Cordwell et al., 2002). Interestingly, among proteins previously known as resistance-related factors (e.g., *mecA, glmM, fmtAB, murE, llm, bla*, and *fem* factors), only FemA protein, which is known as a host-mediated factor essential for methicillin resistance in *S. aureus* (Berger-Bächi et al., 1989), was more highly expressed in methicillin-resistant cells (Cordwell et al., 2002). However, upon growth of both strains in the presence of Triton X-100 (TX-100), a detergent that has been shown to reduce methicillin resistance, no difference on the production of the essential methicillin-resistance factor FemA was detected (Cordwell et al., 2002). Instead, expression levels of stress-related proteins including cold-shock proteins (CspABC) and alkaline-shock protein 23 (Asp23) increased in the methicillin-resistant *S. aureus* strain COL (Cordwell et al., 2002). Notably, the amount of CspB, CspC, and Asp23 proteins was affected in cases of vancomycin and daptomycin antibiotics, despite being down-regulated in the vancomycin-resistant strain and up-regulated in the daptomycin-resistant strain (**Table 7**). This study also showed that three proteins linked to the alternative sigma factor σ^B^, Asp23, anti-anti- σ^B^ factor RsbV, and conserved hypothetical protein SA0772, were also present at significantly higher levels in methicillin-resistant cells (Cordwell et al., 2002). In the presence of TX-100 weakening the methicillin resistance, the comparative proteomic analysis showed that proteins of the σ^B^ and SarA (a regulator of virulence genes) regulons are involved in methicillin resistance of *S. aureus* (Cordwell et al., 2002). The level of SarA protein also increased in vancomycin-resistant and daptomycin-resistant cells (**Table 7**). This study also showed that the stage V sporulation protein G (SpoVG), originally identified in *Bacillus subtilis* as being involved in the formation of the spore cortex (Matsuno and Sonenshein, 1999), was up-regulated in the methicillin-resistant *S. aureus* strain COL. In the non-sporulating *S. aureus*, SpoVG contributes to stimulate capsule synthesis, and was recently shown to regulate a small σ^B^-subregulon comprising mainly excreted virulence factors including the highly up-regulated virulence factor EsxA (Schulthess et al., 2012). Recently, it has been reported that SpoVG was involved in resistance mechanisms to methicillin and glycopeptide (Schulthess et al., 2009). Together with this report, a comparative proteome analysis showed that the expression level of SpoVG increased in strains resistant to methicillin, vancomycin, and daptomycin (**Table 5**), indicating that SpoVG may be involved in resistance mechanisms to other antibiotics as well as methicillin and glycopeptide.

Another report explored proteome profiles of extracellular proteins in methicillin-sensitive and methicillin-resistant *S. aureus* (Enany et al., 2014). They identified some proteins increased in MRSA; Asp23 (10-fold more in MRSA than MSSA), alkyl hydroperoxide reductase subunit C (AhpC) (2-fold), D-lactate dehydrogenase (LdhD) (2-fold), general stress protein 20U (3-fold), L-lactate dehydrogenase (LdhA) (2-fold), pyruvate dehydrogenase E1 component beta subunit (PdhB) (2-fold), superoxide dismutase (SodA) (2-fold), triacylglycerol lipase precursor (LipA) (2-fold), triosephosphate isomerase (TpiA) (2-fold), and universal stress protein family protein (7-fold) (Enany et al., 2014). Notably, among them, most proteins (AhpC, SodA, LdhA, LipA, and TipA) also have altered expression levels in other antibiotic-resistant strains (**Table 7**). In addition, elongation factor G (encoded by the *fusA* gene) was also increased in MRSA. Our analysis showed that PusA is one of the three proteins affected in all four antibiotic-resistant strains (**Table 5**). Although elongation factor G is a major target of fusidic acid which has been used as a topical agent for skin infection and for some systemic infections caused by *S. aureus* (Howden and Grayson, 2006), and had a contribution to fusidic acid resistance mechanisms evolved in MRSA (Koripella et al., 2012), the relationship between elongation factor G and resistance mechanisms of other antibiotics has not yet been identified.

### Vancomycin

There were two studies exploring proteomic profiles in vancomycin-susceptible *S. aureus* (VSSA) and vancomycin-intermediate *S. aureus* (VISA) with a minimal inhibitory concentration (MIC) of 4–8 μg/ml, one study exploring proteomic profiles in VSSA and heterogeneous vancomycin-intermediate *S. aureus* (hVISA) with a vancomycin MIC of ≤2 μg/ml, one study exploring proteomic profiles in VISA and vancomycin-resistant *S. aureus* (VRSA) with MIC of ≥8 μg/ml, one study analyzing global proteomes of vancomycin stress in *S. aureus*, and two studies examining vancomycin-induced proteomes of *Enterococcus faecalis* under vancomycin treatment (Pieper et al., 2006; Scherl et al., 2006; Drummelsmith et al., 2007; Wang et al., 2010; Chen et al., 2013; Hessling et al., 2013; Ramos et al., 2015). Many proteins previously known as resistance-related factors, including VanA, VanB, VanX, and VanR, were also identified in comparative proteomic analyses (Table 2). Scherl et al. (2006) showed that a total of 155 proteins are differentially expressed between two vancomycin-susceptible *S. aureus* strains (MRGR3 and 14-4Rev) and the vancomycin-intermediate *S. aureus* strain 14-4, and most proteins play a role in energy metabolism, cell envelope biosynthesis, protein turnover, amino acids transport, and metabolism, and inorganic ion transport. Genes or gene products known to be involved in resistance mechanisms to different antibiotics, such as PBP 2a (MecA), *O*-nucleotidyltransferase(9) [Ant(9)], UDP-*N*-acetylmuramyl tripeptide synthetase (MurE), and penicillin-binding methicillin resistant-related protein (FmtA), were up-regulated in the VISA strain (Scherl et al., 2006). All of them are involved in peptidoglycan biosynthesis. Levels of many other proteins involved in peptidoglycan metabolism also increased in the VISA strain, such as glycosyltransferase (SgtB) and CHAP (Cysteine, Histidine-dependent Amidohydrolases/Peptidases)-domain amidase (SsaA). SsaA belongs to the CHAP amidase family, members of which such as LysK and LytA have been shown to have D-alanyl-glycyl endopeptidase activity, cleaving between the crossbridge and the stem peptide (Delaune et al., 2011), and protein levels of SsaA were also changed in cases of methicillin and linezolid (**Table 6**), indicating the importance of this protein on peptidoglycan metabolism and antibiotic resistance.

**Table 2:** **Differentially expressed proteins identified by the quantitative proteomic approach: proteins involved in resistance mechanisms**.

**Biological process**	**Protein name**	**Gene**	**Antibiotics**	**Regulation**	**Frequency of difference**	**References**	**Protein description**
Antibiotic inactivation	Bleomycin resistance protein	*ble*	Van	Down	1	Pieper et al., 2006	Inhibition of bleomycin by a direct interaction
	Kanamycin nucleotidyltransferase	*knt*	Van	Down	1	Pieper et al., 2006	Modification of kanamycin
	Vancomycin resistance protein	*vanA*	Van	Up	2	Wang et al., 2010; Ramos et al., 2015	Alteration of the D-Ala-D-Ala dipeptide
	*O*-nucleotidyltransferase (9)	*ant(9)*	Van	Up	1	Scherl et al., 2006	Modification of vancomycin

Tables 2–8: Met, methicillin; Van, vancomycin; Lin, linezolid; Dap, daptomycin; up, up-regulated in antibiotic-resistant strain or under antibiotic treatment; down, down-regulated in antibiotic-resistant strain or under antibiotic treatment.

**Table 3:** **Differentially expressed proteins identified by the quantitative proteomic approach: proteins involved in energy metabolism**.

**Biological process**	**Protein name**	**Gene**	**Antibiotics**	**Regulation**	**Frequency of difference**	**References**	**Protein description**
Energy production and conversion	Pyruvate dehydrogenase E1 component beta subunit	*pdhB*	Met	Up	1	Enany et al., 2014	Acetyl-CoA biosynthetic process from pyruvate
	D-Lactate dehydrogenase	*ldhD*	Met	Up	1	Enany et al., 2014	Pyruvate metabolism
	Formyltetrahydrofolate synthetase	*fhs*	Van	Up	1	Pieper et al., 2006	Glyoxylate and dicarboxylate metabolism and one carbon pool by folate
	Succinyl-CoA synthetase alpha chain	*sucD*	Van	Up	1	Pieper et al., 2006	The citric acid cycle
	Aconitate hydratase	*citB*	Van	Up(down)	1(1)	Pieper et al., 2006; Drummelsmith et al., 2007	The citric acid cycle
	Isocitrate dehydrogenase	*citC*	Van	Up	1	Drummelsmith et al., 2007	The citric acid cycle
	Citrate lyase	*citF*	Van	Up	1	Wang et al., 2010	Acetyl-CoA metabolic process
	ATP synthase γ chain	*atpG*	Van	Up(down)	2(1)	Pieper et al., 2006; Scherl et al., 2006; Wang et al., 2010	ATP formation
	Pyruvate carboxylase	*pycA*	Van	Down	1	Pieper et al., 2006	Anaplerotic reaction
	Malate:quinone oxidoreductase 1	*mqo2*	Van	Up	2	Scherl et al., 2006; Drummelsmith et al., 2007	The citric acid cycle
	2-Dehydro-3-deoxyphosphogluconate aldolase	*eda*	Van	Up	1	Wang et al., 2010	Glycolysis
	Glyceraldehyde-3-phosphate dehydrogenase	*gapA*	Van	Up(down)	1(1)	Wang et al., 2010; Ramos et al., 2015	Glycolysis
	Dihydrolipoamide succinyltransferase	*odhB*	Van	Down	1	Scherl et al., 2006	The citric acid cycle and lysine degradation.
	Glycerophosphoryl diester phosphodiesterase	*glpQ*	Lin	Up	1	Bernardo et al., 2004	Glycerol and glycerophosphodiester degradation
	Lactate oxidase	*lctO*	Lin	Up	1	Feng et al., 2011	Lactate oxidation
	Flavodoxin/nitric oxide synthase	*flaV*	Lin	Up	1	Feng et al., 2011	Flavodoxin biosynthesis
	Gluconate 5-dehydrogenase	*gno*	Lin	Up	1	Feng et al., 2011	Gluconate oxidation
	Phosphoglycolate phosphatase	*gph*	Lin	Up	1	Feng et al., 2011	Glyoxylate and dicarboxylate metabolism
	Enolase (2-phosphoglycerate dehydratase)	*edo*	Dap	Up	1	Fischer et al., 2011	Glycolysis
	Triose-phosphate isomerase	*tpiA*	Met	Up	1	Enany et al., 2014	Glycolysis
			Van	Down	1	Ramos et al., 2015	
	Alcohol dehydrogenase	*adhE*	Met	Down(up)	1(1)	Cordwell et al., 2002; Enany et al., 2014	Fermentation
			Van	Up	2	Drummelsmith et al., 2007; Wang et al., 2010	
	Alcohol dehydrogenase	*adhP*	Met	Up	1	Enany et al., 2014	Fermentation
			Lin	Up	1	Feng et al., 2011	
	2,3-Bisphosphoglycerate-dependent phosphoglycerate mutase	*gpmA*	Van	Up(down)	2(1)	Scherl et al., 2006; Drummelsmith et al., 2007; Chen et al., 2013	Glycolysis
			Lin	Up	1	Feng et al., 2011	
	Nitrate reductase α chain	*narG*	Van	Down	1	Pieper et al., 2006	Anaerobic respiration
			Lin	Down	1	Fischer et al., 2011	
	Phosphoglycerate kinase	*pgk*	Van	Down	2	Pieper et al., 2006; Scherl et al., 2006	Glycolysis
			Lin	Up	1	Feng et al., 2011	
	Phosphopyruvate hydratase	*eno*	Van	Up	1	Scherl et al., 2006	Glycolysis
			Dap	Up	1	Fischer et al., 2011	
	Succinate dehydrogenase flavoprotein subunit	*sdhA*	Van	Up	1	Scherl et al., 2006	The citric acid cycle
			Dap	Down	1	Fischer et al., 2011	
	Pyruvate dehydrogenase α subunit	*pdhA*	Van	Up	1	Wang et al., 2010	Acetyl-CoA biosynthetic process from pyruvate
			Dap	Up	1	Fischer et al., 2011	
	Citrate synthase II	*citZ*	Van	Up	1	Drummelsmith et al., 2007	The citric acid cycle
			Dap	Down	1	Fischer et al., 2011	
	Succinyl-CoA synthetase β chain	*sucC*	Van	Up	2	Pieper et al., 2006; Drummelsmith et al., 2007	The citric acid cycle
			Dap	Down	1	Fischer et al., 2011	
	Aminoethyltransferase	*gcvT*	Van	Up	1	Pieper et al., 2006	Glycine cleavage
			Dap	Up	1	Fischer et al., 2011	
	Glyceraldehyde-3-phosphate dehydrogenase 1	*gapA*	Lin	Up	1	Feng et al., 2011	Glycolysis
			Dap	Up	1	Fischer et al., 2011	
	L-Lactate dehydrogenase	*ldhA*	Met	Up	1	Enany et al., 2014	Fermentation
			Van	Down(up)	2(1)	Pieper et al., 2006; Scherl et al., 2006; Wang et al., 2010	
			Dap	Up	1	Fischer et al., 2011	
	Fructose-bisphosphate aldolase	*fba*	Van	Up	2	Wang et al., 2010; Ramos et al., 2015	Glycolysis
			Lin	Up	1	Feng et al., 2011	
			Dap	Up	1	Fischer et al., 2011	
	Acetate kinase	*ackA*	Met	Up	1	Enany et al., 2014	Fermentation
			Van	Up(down)	1(1)	Scherl et al., 2006; Drummelsmith et al., 2007	
			Dap	Up	1	Fischer et al., 2011	
	Glucose-6-phosphate isomerase	*pgi*	Van	Down(up)	1(1)	Pieper et al., 2006; Scherl et al., 2006	Glycolysis
			Lin	Up	1	Feng et al., 2011	
			Dap	Up	1	Fischer et al., 2011	
Carbohydrate transport and metabolism	ABC transporter, ATP binding protein	*stpC*	Van	Down	1	Drummelsmith et al., 2007	Carbohydrate transport
	PTS transport system, fructose-specific IIABC component	*fruA*	Van	Down	1	Drummelsmith et al., 2007	Fructose transport
	ABC transporter, ATP binding protein	*vraD*	Van	Up	1	Drummelsmith et al., 2007	Bacitracin tansport
	Phosphoglycerate mutase 1	*pgm*	Van	Up	1	Wang et al., 2010	The breakdown of glycogen and metabolism of galactose and maltose
	2,3-Bisphosphoglycerate-independent phosphoglycerate mutase	*gpmI*	Van	Up	1	Drummelsmith et al., 2007	Carbohydrate degradation
	Glycerol kinase	*glpK*	Van	Up	1	Drummelsmith et al., 2007	Carbohydrate degradation
	Lactose PTS system repressor	*fruR*	Lin	Up	1	Feng et al., 2011	Lactose transport
	Glucosamine-6-phosphate isomerase	*nagB*	Lin	Up	1	Feng et al., 2011	Glucosamine metabolism
	Galactose-6-phosphate isomerase	*lacB*	Lin	Up	1	Feng et al., 2011	Galactose metabolism
	Tagatose-6-phosphate kinase	*lacC*	Lin	Up	1	Feng et al., 2011	Tagatose metabolism
	Tagatose 1,6-diphosphate aldolase	*lacD*	Lin	Up	1	Feng et al., 2011	Tagatose metabolism
	β-N-acetylhexosaminidase	*strH*	Lin	Up	1	Feng et al., 2011	Hexosamine metabolism
	β-galactosidase	*bgaA*	Lin	Up	1	Feng et al., 2011	Lactose metabolism
	PTS system transporter subunit IIB	*spr0563*	Lin	Up	1	Feng et al., 2011	Carbohydrate transport
	PTS system transporter subunit IIA	*spr0562*	Lin	Up	1	Feng et al., 2011	Carbohydrate transport
	PTS system transporter subunit IIB	*spr0060*	Lin	Up	1	Feng et al., 2011	Carbohydrate transport
	Fructokinase	*scrK*	Lin	Up	1	Feng et al., 2011	Fructose metabolism
	Glucokinase	*glcK*	Van	Down	1	Scherl et al., 2006	Glucose metabolism
			Lin	Up	1	Feng et al., 2011	
	Catabolite control protein A	*ccpA*	Met	Down	1	Cordwell et al., 2002	Carbon catabolite repression
			Van	Up	1	Wang et al., 2010	
			Lin	Up	1	Feng et al., 2011	

**Table 4:** **Differentially expressed proteins identified by the quantitative proteomic approach: proteins involved in amino acid, nucleotide, coenzyme, and inorganic ion metabolisms**.

**Biological process**	**Protein name**	**Gene**	**Antibiotics**	**Regulation**	**Frequency of difference**	**References**	**Protein description**
Amino acid transport and metabolism	Threonine deaminase	*ilvA*	Met	Down	1	Cordwell et al., 2002	Threonine metabolism
	Aspartate kinase	*lysC*	Van	Up	1	Hessling et al., 2013	Biosynthesis of lysine, methionine, threonine
	Aspartate semialdehyde dehydrogenase	*Asd*	Van	Up	1	Hessling et al., 2013	Biosynthesis of lysine, methionine, threonine
	Dihydrodipicolinate synthase	*dapA*	Van	Up	1	Hessling et al., 2013	Biosynthesis of lysine
	Tetrahydrodipicolinate acetyltransferase	*dapD*	Van	Up	1	Hessling et al., 2013	Biosynthesis of lysine
	Diaminopimelate decarboxylase	*lysA*	Van	Up	1	Hessling et al., 2013	Biosynthesis of lysine
	Arginine deiminase	*arcA*	Van	Down	1	Ramos et al., 2015	Arginine metabolism
	4-Hydroxy-tetrahydrodipicolinate reductase	*dapB*	Van	Up	1	Hessling et al., 2013	Biosynthesis of lysine
	Cystathionine β-lyase	*metC*	Van	Up	1	Wang et al., 2010	Biosynthesis of methionine
	Cystathionine γ-synthase	*metB*	Van	Up	1	Drummelsmith et al., 2007	Biosynthesis of methionine
	Chorismate mutase	*aroA*	Van	Down	1	Drummelsmith et al., 2007	Biosynthesis of aromatic amino acids
	Urease α subunit	*ureC*	Van	Up	1	Drummelsmith et al., 2007	Urea metabolism
	Urease accessory protein	*ureE*	Van	Up	2	Scherl et al., 2006; Drummelsmith et al., 2007	Urea metabolism
	Branched-chain amino acids aminotransferase	*ilvE*	Van	Down	2	Pieper et al., 2006; Scherl et al., 2006	Biosynthesis of branched-chain amino acids
	Aetylornithine aminotransferase 2	*argD*	Van	Up	1	Pieper et al., 2006	Biosynthesis of lysine
	Amino acid ABC transporter amino acid-binding protein	*glnH*	Lin	Up	1	Feng et al., 2011	Glutamine transport
	Tryptophan synthase subunit α	*trpA*	Lin	Down	1	Feng et al., 2011	Biosynthesis of aromatic amino acids
	Asparagine synthetase	*asnA*	Lin	Down	1	Feng et al., 2011	Biosynthesis of asparagine
	Aminotransferase	*aspC*	Lin	Up	1	Feng et al., 2011	Biosynthesis of lysine, methionine, threonine
	Carbamate kinase	*arcC*	Van	Down	2	Scherl et al., 2006; Ramos et al., 2015	Allantoin degradation
			Lin	Up	1	Feng et al., 2011	
	1-Pyrroline-5-carboxylate dehydrogenase	*rocA*	Van	Up	1	Scherl et al., 2006	Proline degradation
			Dap	Down	1	Fischer et al., 2011	
	Serine hydroxymethyltransferase	*glyA*	Van	Down	1	Drummelsmith et al., 2007	Biosynthesis of glycine
			Dap	Up	1	Fischer et al., 2011	
	Glutamine synthetase type 1	*glnA*	Lin	Down	1	Feng et al., 2011	Nitrogen assimilation
			Dap	Down	1	Fischer et al., 2011	
Nucleotide transport and metabolism	Bifunctional pyrimidine regulatory protein /uracil phosphoribosyltransferase	*pyrR*	Met	Down	1	Cordwell et al., 2002	Salvage pathways of pyrimidine ribonucleotides
	Purine nucleoside phosphorylase	*deoD*	Van	Down	1	Pieper et al., 2006	Guanosine nucleotides *de novo* biosynthesis
	Putative purine biosynthesis protein	*purS*	Van	Up	1	Pieper et al., 2006	Purine biosynthesis
	Adenylosuccinate synthetase	*purA*	Van	Up	1	Pieper et al., 2006	Purine biosynthesis
	Phosphoribosylaminoimidazole carboxylase	*purK*	Van	Up	1	Pieper et al., 2006	Purine biosynthesis
	Phosphoribosylglycinamidine synthase I	*purQ*	Van	Up	1	Pieper et al., 2006	Purine biosynthesis
	Phosphoribosylglycinamidine synthase II	*purL*	Van	Up	1	Pieper et al., 2006	Purine biosynthesis
	Phosphoribosylglycinamide formyltransferase	*purN*	Van	Up	1	Pieper et al., 2006	Purine biosynthesis
	Phosphoribosykaminoimidazole carboxylase, catalytic subunit	*purE*	Van	Up	1	Pieper et al., 2006	Purine biosynthesis
	GMP synthase	*guaA*	Van	Down	2	Scherl et al., 2006; Ramos et al., 2015	Biosynthesis of guanosine nucleotides
	The *pur* operon repressor	*purR*	Lin	Up	1	Feng et al., 2011	Purine biosynthesis
	Ribose-phosphate pyrophosphokinase	*prs*	Van	Up	2	Scherl et al., 2006; Drummelsmith et al., 2007	Purine biosynthesis
			Lin	Up	1	Feng et al., 2011	
	Amidophosphoribosyltransferase	*purF*	Van	Up	1	Pieper et al., 2006	Purine biosynthesis
			Dap	Up	1	Fischer et al., 2011	
	Phosphoribosylamine-glycine ligase	*purD*	Van	Up	1	Pieper et al., 2006	Purine biosynthesis
			Dap	Down	1	Fischer et al., 2011	
	Phosphoribosylglycinamidine cyclo-ligase	*purM*	Van	Up	1	Pieper et al., 2006	Purine biosynthesis
			Dap	Down	1	Fischer et al., 2011	
	GMP reductase	*guaC*	Van	Up	1	Pieper et al., 2006	The purine salvage pathway
			Dap	Down	1	Fischer et al., 2011	
	Dihydroorotase	*pyrC*	Van	Down	1	Drummelsmith et al., 2007	Pyrimidine biosynthesis
			Dap	Down	1	Fischer et al., 2011	
	Carbamoyl phosphate synthase large subunit	*carB*	Van	Down	1	Scherl et al., 2006	Pyrimidine biosynthesis
			Dap	Up	1	Fischer et al., 2011	
	Phosphoribosylaminoimidazole-succinocarboxamide synthase	*purC*	Van	Up	1	Pieper et al., 2006	Purine biosynthesis
			Dap	Up	1	Fischer et al., 2011	
	Adenylosuccinate lyase	*purB*	Met	Up	1	Enany et al., 2014	Purine biosynthesis
			Van	Up	2	Pieper et al., 2006	
			Dap	Up	1	Fischer et al., 2011	
	Bifunctional purine biosynthesis protein	*purH*	Van	Up	1	Pieper et al., 2006	Purine biosynthesis
			Lin	Down	1	Feng et al., 2011	
			Dap	Up	1	Fischer et al., 2011	
	Uracil phosphoribosyltransferase	*upp*	Van	Down	2	Scherl et al., 2006; Drummelsmith et al., 2007	Salvage pathways of pyrimidine ribonucleotides
			Lin	Down	1	Feng et al., 2011	
			Dap	Up	1	Fischer et al., 2011	
Coenzyme transport and metabolism	3-Hydroxy-3-methylglutaryl-CoA synthase	*mvaS*	Met	Up	1	Cordwell et al., 2002	Isoprenoid biosynthesis
	Thiamin-biosynthesis protein	*thiL*	Met	Up	1	Cordwell et al., 2002	Thiamin biosynthesis
	δ-aminoevulinic acid dehydratase	*hemB*	Van	Down	1	Pieper et al., 2006	Heme biosynthesis
	Molybdopterin converting factor subunit 2	*moaE*	Van	Down	1	Pieper et al., 2006	Molybdenum cofactor biosynthesis
	2-Dehydropantoate 2-reductase	*panE*	Van	Down	1	Drummelsmith et al., 2007	Pantothenate and coenzyme A biosynthesis
	6-Pyruvoyl tetrahydrobiopterin synthase	*ptpS*	Van	Up	1	Drummelsmith et al., 2007	Tetrahydrobiopterin biosynthesis
	Phosphopantetheine adenylyltransferase	*coaD*	Van	Up	1	Drummelsmith et al., 2007	Pantothenate and coenzyme A biosynthesis
	Coenzyme A disulfide reductase	*cdr*	Van	Down	1	Scherl et al., 2006	Pantothenate and coenzyme A biosynthesis
	Hydroxyethylthiazole kinase	*thiM*	Lin	Up	1	Feng et al., 2011	Thiamin biosynthesis
	3-Methyl-2-oxobutanoate hydroxymethyltransferase	*panB*	Met	Up	1	Enany et al., 2014	Pantothenate and coenzyme A biosynthesis
			Van	Down(up)	1(1)	Drummelsmith et al., 2007; Wang et al., 2010	
	Bifunctional 5,10-methylene-tetrahydrofolate dehydrogenase/5,10-methylene-tetrahydrofolate cyclohydrolase	*folD*	Van	Up	1	Pieper et al., 2006	*N*^10^-formyl-tetrahydrofolate biosynthesis
			Dap	Up	1	Fischer et al., 2011	
	6,7-Dimethyl-8-ribityllumazine synthase	*ribH*	Lin	Up	1	Feng et al., 2011	Flavin biosynthesis
			Dap	Up	1	Fischer et al., 2011	
	Pyridoxal biosynthesis lyase	*pdxS*	Lin	Down	2	Drummelsmith et al., 2007; Feng et al., 2011	Pyridoxal 5′-phosphate biosynthesis.
			Dap	Up	1	Fischer et al., 2011	
Inorganic ion transport and metabolism	ABC protein/substrate binding protein subunit—metal ion transport	*lmb*	Lin	Up	1	Feng et al., 2011	Metal ion transport
	Non-heme iron-containing ferritin	*dpr*	Lin	Up	1	Feng et al., 2011	Iron tansport
	Iron-compound ABC transporter permease	*fatD*	Lin	Up	1	Feng et al., 2011	Iron tansport
	Lipoprotein similar to streptococcal adhesin	*psaA*	Lin	Up	1	Feng et al., 2011	Manganese transport; pneumococcal attachment
			Dap	Down	1	Fischer et al., 2011	

They also identified several regulatory systems contributing to the VISA phenotype, such as the two-component system (VraSR) regulating expression of a set of genes involved in the cell wall biosynthesis or degradation (Boyle-Vavra et al., 2013), the signal transduction protein TRAP acting on quorum sensing (Gov et al., 2004), the DivIVA protein known to regulate cell division in *B. subtilis* (Perry and Edwards, 2004), and putative transcription factors SA2296 and SarH1. VraSR (vancomycin resistance associated regulator) was up-regulated under vancomycin treatment (Kuroda et al., 2003) and in the VISA strain when compared with an isogenic vancomycin-susceptible strain (Kuroda et al., 2000). In addition, inactivation of the *vraSR* gene increased vancomycin susceptibility (Kuroda et al., 2003). Interestingly, VraSR was also induced by other antibiotic classes that target the cell wall, including β-lactam (Gardete et al., 2006; Yin et al., 2006), mersacidin (Sass et al., 2008), certain cationic peptides (Pietiäinen et al., 2009), and daptomycin (Muthaiyan et al., 2008). Inactivation of the *vraSR* gene attenuates resistance to various antibiotics, such as vancomycin (Kuroda et al., 2003; Gardete et al., 2006), daptomycin (Mehta et al., 2012), and β-lactams (Kuroda et al., 2003; Boyle-Vavra et al., 2006; Gardete et al., 2006). The expression of many genes, such as *ctpA, drp35, fmtA, opuD, pbp2, prsA, sgtB*, and *vraX*, is regulated by VraSR (Utaida et al., 2003; McAleese et al., 2006; Dengler et al., 2011). Among them, FmtA is typically known as a factor involved in methicillin-resistant phenotype of *S. aureus* (Fan et al., 2007), and PrsA (foldase precursor) was recently reported to be involved in both glycopeptide and oxacillin resistance in *S. aureus* (Jousselin et al., 2012). Similarly, at three independent studies of comparative proteomic analysis, it has been proven that the expression level of PrsA is up-regulated in VISA when compared with VSSA (Table 5), indicating that proteomic studies can support the identification of targets involved in antibiotic resistance. They also identified another important protein VraX (a hypothetical protein which encodes a 55-amino acids protein) differentially expressed between vancomycin-susceptible *S. aureus* strains and the vancomycin-intermediate *S. aureus* strain 14-4 (Scherl et al., 2006). This gene was up-regulated by multiple cell wall and/or membrane active compounds (bacitracin, d-cycloserine, oxacillin, tunicamycin, flavomycin, fosfomycin, teicoplanin, vancomycin, daptomycin, lysostaphin, epicatechin gallate, ranalexin, and antimicrobial peptides) (Utaida et al., 2003; Pietiäinen et al., 2009; Dengler et al., 2011; Cuaron et al., 2013). The *vraX* gene belongs to the *vra* operon together with the *vraA* gene encoding for a long chain fatty acid-CoA ligase, which was up-regulated in the VISA. Additionally, this gene seems to be involved in resistance mechanism to vancomycin (Hanaki et al., 1998; Buntaran et al., 2013). Finally, stress-related proteins such as proteinases (CtpA), methionine sulfoxide reductase A (MsrA2), and the methionine sulfoxide reductase regulator MsrR, were over-expressed in the vancomycin-intermediate *S. aureus* strain 14-4 (Scherl et al., 2006). In other studies, MsrA2 was also up-regulated in hVISA (Chen et al., 2013).

**Table 5:** **Differentially expressed proteins identified by the quantitative proteomic approach: proteins involved in replication, cell division, transcription, translation, and protein turnover**.

**Biological process**	**Protein name**	**Gene**	**Antibiotics**	**Regulation**	**Frequency of difference**	**References**	**Protein description**
Replication, recombination and repair	Initiation-control protein	*yabA*	Van	Up	1	Ramos et al., 2015	Replication
	Topoisomerase IV subunit B	*parE*	Van	Down	1	Pieper et al., 2006	Replication
	DNA gyrase subunit B	*gyrB*	Van	Down	1	Pieper et al., 2006	Replication
	Single-stranded DNA binding protein	*traM*	Van	Down	1	Pieper et al., 2006	Replication
	Formamidopyrimidine-DNA glycosylase	*mutM*	Lin	Up	1	Feng et al., 2011	DNA repair
	Single-stranded DNA-binding protein	*ssbB*	Lin	Down	1	Feng et al., 2011	Replication
	DNA-entry nuclease	*endA*	Lin	Up	1	Feng et al., 2011	DNA repair
	Recombinase A	*recA*	Van	Up	1	Wang et al., 2010	Recombination
			Dap	Up	1	Fischer et al., 2011	
	Endonuclease IV	*nfo*	Van	Up	1	Wang et al., 2010	DNA repair
			Dap	Down	1	Fischer et al., 2011	
Sporulation and cell division	Anti-anti-σ^B^ factor	*rsbV*	Met	Up	1	Cordwell et al., 2002	Sporulation
	Cell division protein	*mraZ*	Van	Up	1	Pieper et al., 2006	Cell division
	Cell division protein	*divIVA*	Van	Up	1	Scherl et al., 2006	Cell division
	Cell division protein	*ftsZ*	Van	Up	1	Wang et al., 2010	Cell division
			Dap	Up	1	Fischer et al., 2011	
	Regulatory protein SpoVG	*spoVG*	Met	Up	1	Cordwell et al., 2002	Sporulation
			Van	Up	1	Pieper et al., 2006	
			Dap	Up	1	Fischer et al., 2011	
Transcription	DNA-directed RNA polymerase subunit delta	*rpoE*	Lin	Up	1	Feng et al., 2011	
	Transcription elongation factor	*greA*	Van	Down	1	Pieper et al., 2006	Trnascription
			Lin	Up	1	Feng et al., 2011	
Translation, ribosomal structure and biogenesis	5-methylaminomethyl-2-thiouridylate)-methyltransferase	*trmU*	Van	Up	1	Wang et al., 2010	tRNA modification
	16S rRNA processing protein	*rimM*	Lin	Down	1	Feng et al., 2011	Ribosome maturation
	Acetyltransferase	*rimL*	Lin	Up	1	Feng et al., 2011	Ribosome modification
	Methionine aminopeptidase	*map*	Lin	Up	1	Feng et al., 2011	Amino-terminal maturation
	Ribosomal subunit interface protein	*spr2011*	Lin	Up	1	Feng et al., 2011	Ribosome regulation
	Ribosomal protein S4	*rpsD*	Dap	Up	1	Fischer et al., 2011	Ribosomal subunit protein
	Ribosomal protein S10	*rpsJ*	Met	Up	1	Enany et al., 2014	Ribosomal subunit protein
			Dap	Up	1	Fischer et al., 2011	
	Ribosomal protein S13	*rpsM*	Met	Up	1	Enany et al., 2014	Ribosomal subunit protein
			Dap	Up	1	Fischer et al., 2011	
	Ribosomal protein S3	*rpsC*	Met	Up	1	Enany et al., 2014	Ribosomal subunit protein
			Dap	Up	1	Fischer et al., 2011	
	Ribosomal protein L7/L12	*rplL*	Met	Up	1	Enany et al., 2014	Ribosomal subunit protein
			Dap	Up	1	Fischer et al., 2011	
	Translational initiation factor IF-2	*infB*	Van	Down	1	Pieper et al., 2006	Translation
			Dap	Up	1	Fischer et al., 2011	
	Essential GTPase	*era*	Van	Down	1	Pieper et al., 2006	Ribosome maturation
			Dap	Up	1	Fischer et al., 2011	
	Ribosomal protein L2	*rplB*	Van	Down	1	Hessling et al., 2013	Ribosomal subunit protein
			Dap	Up	1	Fischer et al., 2011	
	GTP-binding protein	*engA*	Van	Down	1	Scherl et al., 2006	Ribosome maturation
			Dap	Up	1	Fischer et al., 2011	
	Ribosomal protein S18	*rpsR*	Lin	Up	1	Feng et al., 2011	Ribosomal subunit protein
			Dap	Up	1	Fischer et al., 2011	
	Ribosomal protein L3	*rplC*	Lin	Up	1	Bernardo et al., 2004	Ribosomal subunit protein
			Dap	Up	1	Fischer et al., 2011	
	Ribosomal protein L27	*rpmA*	Lin	Up	1	Bernardo et al., 2004	Ribosomal subunit protein
			Dap	Up	1	Fischer et al., 2011	
	Ribosomal protein L22	*rplV*	Lin	Up	1	Bernardo et al., 2004	Ribosomal subunit protein
			Dap	Up	1	Fischer et al., 2011	
	Ribosomal protein S9	*rplI*	Lin	Up	1	Bernardo et al., 2004	Ribosomal subunit protein
			Dap	Up	1	Fischer et al., 2011	
	Ribosomal protein L15	*rplO*	Lin	Up	1	Bernardo et al., 2004	Ribosomal subunit protein
			Dap	Up	1	Fischer et al., 2011	
	Ribosomal protein L13	*rplM*	Lin	Up	1	Bernardo et al., 2004	Ribosomal subunit protein
			Dap	Up	1	Fischer et al., 2011	
	Ribosomal protein L4	*rplD*	Lin	Up	1	Bernardo et al., 2004	Ribosomal subunit protein
			Dap	Up	1	Fischer et al., 2011	
	Ribosomal protein L1	*rplA*	Lin	Up	2	Bernardo et al., 2004; Feng et al., 2011	Ribosomal subunit protein
			Dap	Up	1	Fischer et al., 2011	
	30S ribosomal protein S1	*rpsA*	Met	Up	1	Enany et al., 2014	Ribosomal subunit protein
			Van	Down	1	Drummelsmith et al., 2007	
			Dap	Up	1	Fischer et al., 2011	
	Ribosomal protein L14	*rplN*	Met	Up	1	Enany et al., 2014	Ribosomal subunit protein
			Van	Down	1	Hessling et al., 2013	
			Dap	Up	1	Fischer et al., 2011	
	Ribosomal protein L21	*rplU*	Met	Up	1	Enany et al., 2014	Ribosomal subunit protein
			Lin	Up	2	Bernardo et al., 2004; Feng et al., 2011	
			Dap	Up	1	Fischer et al., 2011	
	Ribosomal protein S6	*rpsF*	Met	Up	1	Enany et al., 2014	Ribosomal subunit protein
			Lin	Up	1	Feng et al., 2011	
			Dap	Up	1	Fischer et al., 2011	
	Ribosomal protein L6	*rplF*	Van	Down	1	Hessling et al., 2013	Ribosomal subunit protein
			Lin	Up	1	Bernardo et al., 2004	
			Dap	Up	1	Fischer et al., 2011	
	Ribosomal protein S2	*rpsB*	Van	Up	1	Wang et al., 2010	Ribosomal subunit protein
			Lin	Up(down)	1(1)	Bernardo et al., 2004; Feng et al., 2011	
			Dap	Up	1	Fischer et al., 2011	
	Elongation factor Tu	*tuf*	Van	Down(up)	1(1)	Drummelsmith et al., 2007; Wang et al., 2010	Translation
			Lin	Up	2	Bernardo et al., 2004; Feng et al., 2011	
			Dap	Up	1	Fischer et al., 2011	
	50S ribosomal protein L20	*rplT*	Van	Up	1	Drummelsmith et al., 2007	Ribosomal subunit protein
			Lin	Down	1	Feng et al., 2011	
			Dap	Up	1	Fischer et al., 2011	
	Elongation factor Ts	*tsf*	Van	Down	1	Pieper et al., 2006	Translation
			Lin	Up	1	Feng et al., 2011	
			Dap	Down	1	Fischer et al., 2011	
	Translational elongation factor G	*fusA*	Met	Up	1	Enany et al., 2014	Translation
			Van	Down(up)	1(1)	Drummelsmith et al., 2007; Wang et al., 2010	
			Lin	Up	1	Bernardo et al., 2004	
			Dap	Up	1	Fischer et al., 2011	
Post-translational modification, protein turnover, chaperones	ATP-dependent Clp protease proteolytic subunit	*clpP*	Met	Up	1	Cordwell et al., 2002	Protein degradation
	Preprotein translocase	*secY*	Van	Up	1	Scherl et al., 2006	Protein translocation
	ATP-dependent chaperone protein	*clpB*	Van	Up	1	Hessling et al., 2013	Protein degradation
	Aminopeptidase	*pepS*	Van	Down	1	Drummelsmith et al., 2007	Protein degradation
	Foldase precursor	*prsA*	Van	Up	3	Scherl et al., 2006; Drummelsmith et al., 2007; Hessling et al., 2013	Chaperone
	Chaperone	*groS*	Van	Up	1	Pieper et al., 2006	Chaperone
	Methionine sulfoxide reductase A	*msrA*	Van	Up	2	Scherl et al., 2006; Chen et al., 2013	Protein modification
	Carboxy-terminal processing peptidase	*ctpA*	Van	Up	1	Scherl et al., 2006	Protein processing
	Cell wall-associated serine proteinase precursor	*prtA*	Lin	Up	1	Feng et al., 2011	Protein degradation
	Methionine sulfoxide reductase B	*SA1256*	Met	Up	1	Cordwell et al., 2002	Protein modification
			Van	Up	1	Scherl et al., 2006	
	Glutamyl-aminopeptidase	*pepA*	Van	Up	1	Wang et al., 2010	Protein degradation
			Lin	Up	1	Feng et al., 2011	
	Chaperone	*dnaK*	Van	Up(down)	2(1)	Scherl et al., 2006; Drummelsmith et al., 2007; Wang et al., 2010	Chaperone
			Lin	Up	1	Bernardo et al., 2004	
	Signal peptidase B	*spsB*	Van	Up	2	Scherl et al., 2006; Drummelsmith et al., 2007	Cleavage of signal peptide
			Dap	Down	1	Fischer et al., 2011	
	Peptide methionine sulfoxide reductase regulator	*msrR*	Van	Up	1	Scherl et al., 2006	Protein modification
			Dap	Down	1	Fischer et al., 2011	
	Peptidase	*ftsH*	Van	Up	2	Scherl et al., 2006; Drummelsmith et al., 2007	Protein degradation
			Dap	Down	1	Fischer et al., 2011	

Pieper et al. showed that purine ribonucleotide biosynthesis (PRNBS) pathway enzymes, which are under the control of the PurR regulator, strongly increased in protein abundance in the vancomycin-resistant *S. aureus* strain VP32 having a vancomycin MIC of 32 μg/ml when compared with the vancomycin-intermediate *S. aureus* strain HIP5827 (MIC = 8 μg/ml) (Pieper et al., 2006). Notably, among them, several proteins such as amidophosphoribosyltransferase (PurF), phosphoribosylamine-glycine ligase (PurD), phosphoribosylglycinamidine cyclo-ligase (PurM), phosphoribosylaminoimidazole-succinocarboxamide synthase (PurC), adenylosuccinate lyase (PurB), and bifunctional purine biosynthesis protein (PurH), were also changed in protein abundance in cases of other antibiotics such as daptomycin and linezolid (Table 4). Microarray transcription analysis of clinical VISA isolates already showed that among the 35 genes with increased transcription in vancomycin-resistant *S. aureus* strain VP32 when compared with those of their VISA parent strains HIP5827 and P100, 15 were involved in purine biosynthesis or transport (Mongodin et al., 2003). They hypothesized that increased energy (ATP) is required to generate the thicker cell walls that characterize resistant mutants (Mongodin et al., 2003). However, contrary to these results, other comparative proteomic analyses between vancomycin-susceptible strains and vancomycin-intermediate *S. aureus* strains did not show similar results (Scherl et al., 2006; Drummelsmith et al., 2007; Chen et al., 2013). Therefore, these results imply that VRSA may more efficiently compensate for a fitness cost of antibiotic resistance such as ATP requirement than VISA.

Abundance changes were also found in proteins such as the single-stranded DNA binding protein (TraM), DNA gyrase subunit B (GyrB), and topoisomerase IV subunit B (ParE), which catalyze or influence the fidelity of DNA replication and repair (Table 5). This result is consistent with the increasing evidence that exposure to antibiotics in bacteria leads to increased mutation rates in the genome, to favor their survivals under antibiotic pressure (Napolitano et al., 2000; Friedberg et al., 2002; Pieper et al., 2006). Expression levels of many enzymes involved in energy metabolisms, including L-lactate dehydrogenase (LdhA), glucose-6-phosphate isomerase (Pgi), succinyl-CoA synthetase (SucCD), phosphoglycerate kinase (Pgk), nitrate reductase alpha chain (NarG), and aconitate hydratase (CitB), were also changed. In fact, comparative proteomic analyses show that proteins involved in energy metabolism, protein synthesis, and envelope biogenesis, most frequently exhibit abundance change in antibiotic-resistant strains (Table 3). In many cases, proteins playing a role in energy metabolism were up-regulated in antibiotic-resistant strains (Table 3). This phenomenon may be explained by a prior hypothesis that increased energy (ATP) is required to generate the thicker cell walls or to pump antibiotics out of the cell using efflux pumps. This study also showed the changes of proteins involved in cell envelope biogenesis, such as D-Ala-D-Ala ligase (Ddl), D-Ala-D-Lac ligase (VanA), peptidoglycan hydrolase (LytM), cell division and cell wall biosynthesis protein (MraZ), putative cell wall transglycosylase (SceD), and glucosamine-fructose-6-phosphate aminotransferase (GlmS) (Pieper et al., 2006).

Similar to prior reports, Drummelsmith et al. showed the high level inductions of cell wall metabolism-related proteins such as MecA, LytM, GlmS, and SceD in the VISA type strain Mu50 when compared with the vancomycin-sensitive strain CMRSA-2 (Drummelsmith et al., 2007). In particular, they selected SceD for further study based on its high level of induction (approximately 16-fold) in VISA, and relative *sceD* mRNA expression levels were compared between 25 VSSA and VISA clinical isolates by real-time RT-PCR (Drummelsmith et al., 2007). The *sceD* mRNA was significantly induced in all VISA isolates relative to all VSSA strains, and they suggest that SceD expression level could serve as a molecular diagnostic marker for the rapid detection of VISA (Drummelsmith et al., 2007). Interestingly, SceD was also up-regulated in both daptomycin-resistant (Song et al., 2013) and linezolid-resistant strains (Bernardo et al., 2004), suggesting the importance of this protein in antibiotic resistance. They also identified other proteins involved in cell envelope metabolism as a highly up-regulated protein in VISA; UDP-GlcNAc 1-carboxyvinyltransferase 1 (MurA), bifunctional autolysin (Atl), immunodominant antigen A (IsaA), UDP-glucose/GDP-mannose dehydrogenase (CapO), and UDP-*N*-acetyltalosamine 2-epimerase (CapG) (Table 6). Among them, IsaA was also up-regulated in VISA at other two studies (Scherl et al., 2006; Chen et al., 2013). In addition, its expression level increased in both methicillin-resistant and daptomycin-resistant strains (Cordwell et al., 2002; Fischer et al., 2011), and decreased in linezolid-resistant strains (Bernardo et al., 2004), suggesting the importance of this protein. The housekeeping protein IsaA is a highly immunogenic, non-covalently cell wall-bound lytic transglycosylase that is co-regulated with a glycylglycine endopeptidase LytM (Stapleton et al., 2007; Lorenz et al., 2011). *S. aureus* has two putative peptidoglycan hydrolases, IsaA and SceD, and SceD can compensate for the loss of IsaA (Stapleton et al., 2007). The fact that both peptidoglycan hydrolases (IsaA and SceD) are involved in antibiotic resistance strongly indicates the importance of cell wall dynamics in antibiotic resistance mechanism.

**Table 6:** **Differentially expressed proteins identified by the quantitative proteomic approach: proteins involved in envelope biogenesis**.

**Biological process**	**Protein name**	**Gene**	**Antibiotics**	**Regulation**	**Frequency of difference**	**References**	**Protein description**
Cell wall, membrane, envelope biogenesis	Acyl carrier protein	*acpP*	Met	Up	1	Enany et al., 2014	Membrane biosynthesis
	2-C-methyl-D-erythritol 4-phosphate cytidylyltransferase	*ispD*	Met	Up	1	Enany et al., 2014	Isoprenoid biosynthesis
	Capsular polysaccharide synthesis enzyme	*cap8H*	Van	Up	1	Scherl et al., 2006	Capsular polysaccharide biosynthesis
	Isopentenyl-diphosphate delta-isomerase	*fni*	Van	Down	1	Drummelsmith et al., 2007	Biosynthesis of isoprenoids
	Malonyl CoA-ACP transacylase	*fabD*	Van	Down	1	Scherl et al., 2006	Fatty acid biosynthesis
	Teichoic acid biosynthesis protein B	*tagB*	Van	Up	1	Scherl et al., 2006	Teichoic acid biosynthesis
	Capsular polysaccharide synthesis protein Cap5D	*capD*	Van	Up	1	Scherl et al., 2006	Capsular polysaccharide biosynthesis
	Capsular polysaccharide synthesis protein Cap5M	*capM*	Van	Up	2	Scherl et al., 2006; Hessling et al., 2013	Capsular polysaccharide biosynthesis
	Capsular polysaccharide synthesis protein Cap5A	*capA*	Van	Up	1	Scherl et al., 2006	Capsular polysaccharide biosynthesis
	UDP-glucose/GDP-mannose dehydrogenase	*capO*	Van	Up	1	Drummelsmith et al., 2007	Capsular polysaccharide biosynthesis
	D-alanine-d-alanine ligase	*ddl*	Van	Up	3	Pieper et al., 2006; Hessling et al., 2013; Ramos et al., 2015	Peptidoglycan biosynthesis
	D-alanine-d-alanine dipeptidase	*ddpX*	Van	Up	1	Ramos et al., 2015	Peptidoglycan biosynthesis
	D-alanine-d-lactate dipeptidase	*vanX*	Van	Up	1	Wang et al., 2010	Peptidoglycan biosynthesis
	D-alanine-d-lactate ligase	*vanB*	Van	Up	1	Wang et al., 2010	Peptidoglycan biosynthesis
	Surface determinant protein A	*isdA*	Van	Down	2	Scherl et al., 2006; Drummelsmith et al., 2007	
	UDP-N-acetyltalosamine 2-epimerase	*capG*	Van	Up	1	Drummelsmith et al., 2007	Capsular polysaccharide biosynthesis
	Glycosyltransferase	*sgtB*	Van	Up	1	Scherl et al., 2006	
	Penicillin binding protein 2A	*mecA*	Van	Up	2	Scherl et al., 2006; Drummelsmith et al., 2007	Peptidoglycan biosynthesis
	Peptidoglycan hydrolase	*lytM*	Van	Up	2	Pieper et al., 2006; Drummelsmith et al., 2007	Peptidoglycan degradation
	UDP-*N*-acetylmuramyl tripeptide synthetase	*murE*	Van	Up	1	Scherl et al., 2006	Peptidoglycan biosynthesis
	Enoyl-CoA hydratase	*phaB*	Lin	Down	1	Feng et al., 2011	Fatty acid β-oxidation
	3-Ketoacyl-ACP reductase	*fabG*	Lin	Down	1	Feng et al., 2011	Fatty acids biosynthesis
	Acetyl-CoA carboxylase biotin carboxyl carrier protein subunit	*accB*	Lin	Down	1	Feng et al., 2011	Fatty acid biosynthesis
	Acetyl-CoA carboxylase subunit α	*accA*	Lin	Down	1	Feng et al., 2011	Fatty acid biosynthesis
	Control of cell shape; membrane-associated protein	*mreBH*	Dap	Up	1	Wecke et al., 2009	Control of cell shape
	Squalene synthase	*crtN*	Dap	Down	1	Fischer et al., 2011	Isoprenoid biosynthesis
	Glucosamine-fructose-6-phosphate aminotransferase	*glmS*	Van	Up	2	Pieper et al., 2006; Drummelsmith et al., 2007	Peptidoglycan biosynthesis
			Dap	Up	1	Fischer et al., 2011	
	UDP-GlcNAc 1-carboxyvinyltransferase 1	*murA*	Van	Up	1	Drummelsmith et al., 2007	Peptidoglycan biosynthesis
			Dap	Down	1	Fischer et al., 2011	
	3-Oxoacyl-ACP synthase II	*fabF*	Van	Up	2	Scherl et al., 2006; Wang et al., 2010	Fatty acid biosynthesis
			Dap	Up	1	Fischer et al., 2011	
	Bifunctional N-acetylglucosamine-1-phosphate uridyltransferase/glucosamine-1-phosphate acetyltransferase	*glmU*	Lin	Up	1	Feng et al., 2011	Peptidoglycan biosynthesis
			Dap	Up	1	Fischer et al., 2011	
	CHAP (Cysteine, Histidine-dependent Amidohydrolases/Peptidases)-domain amidase	*ssaA*	Met	Up	1	Cordwell et al., 2002	Peptidoglycan degradation
			Van	Up	2	Scherl et al., 2006; Drummelsmith et al., 2007	
			Lin	Down	1	Bernardo et al., 2004	
	Triacylglycerol lipase precursor	*lipA*	Met	Up	1	Enany et al., 2014	Lipoate biosynthesis
			Lin	Up	1	Bernardo et al., 2004	
			Dap	Down	1	Fischer et al., 2011	
	Aminoacyltransferase	*femA*	Met	Up	1	Cordwell et al., 2002	Peptidoglycan biosynthesis
			Van	Up	2	Scherl et al., 2006; Hessling et al., 2013	
			Dap	Up	1	Fischer et al., 2011	
	Penicillin-binding protein 1	*pbpA*	Met	Up	1	Cordwell et al., 2002	Peptidoglycan biosynthesis
			Van	Up	1	Scherl et al., 2006	
			Dap	Down	1	Fischer et al., 2011	
	Hydroxymyristoyl ACP dehydratase	*fabZ*	Met	Up	1	Enany et al., 2014	Fatty acid biosynthesis
			Van	Down	1	Drummelsmith et al., 2007	
			Lin	Down	1	Feng et al., 2011	
			Dap	Down	1	Fischer et al., 2011	

To identify the resistance mechanisms of hVISA with a vancomycin MIC of ≤2 μg/ml, Chen et al. compared proteomic profiles of six pairs of isogenic hVISA and VSSA strains and unrelated hVISA (*n* = 24) and VSSA stains (*n* = 30) (Chen et al., 2013). They identified five proteins up-regulated in the hVISA strains; IsaA, MsrA, Asp32, 2,3-bisphosphoglycerate-dependent phosphoglycerate mutase (GpmA), and AhpC. Consistent with this result, MsrA was up-regulated in a prior study using comparative proteomics (Scherl et al., 2006) and in the DNA microarray study, and the *msrA* gene was also over-expressed in VISA strains (Cui et al., 2005). MsrA, catalyzing the reversible oxidation-reduction of methionine sulfoxide to methionine, has a key function as a repair enzyme for proteins inactivated by oxidation (Chen et al., 2013). The *msrA* gene is highly induced by cell wall-active antibiotics, such as oxacillin and vancomycin (Chen et al., 2013). The increased level of MsrA can enhance peptidoglycan biosynthesis which results in cell wall thickening, and gene knockout of the *msrA* gene weakened vancomycin and β-lactam resistance of *S. aureus* strains (Cui et al., 2005). In addition, MsrA is involved in virulence in several bacteria (Sasindran et al., 2007). Taken together, these observations suggest the important role of methionine sulfoxide in antibiotic resistance. Although in other studies, the abundance of GpmA, which plays a physiological role in glycolysis, has been reported to be changed in VISA (Table 3), its exact role in antibiotic resistance has not been determined. AhpC, an alkyl hydroperoxide reductase subunit C, plays an important role in oxidative-stress resistance of *S. aureus* (Cosgrove et al., 2007). Interestingly, it was reported that AhpC is up-regulated in strains resistant to methicillin, vancomycin, and daptomycin antibiotics (Table 7). However, up to now, there is no report investigating the direct role of AhpC in antibiotic resistance. It is noteworthy that several proteins involved in oxidative-stress resistance, such as AhpC, SodA, catalase (KatA), and superoxide dismutase (SodM), show the abundance change of proteins in antibiotic-resistant strains (Table 7), and in most cases, their expression is up-regulated. In spite of these interesting results, the relationship between these proteins and antibiotic resistance was not determined.

**Table 7:** **Differentially expressed proteins identified by the quantitative proteomic approach: proteins involved in stress response**.

**Biological process**	**Protein name**	**Gene**	**Antibiotics**	**Regulation**	**Frequency of difference**	**References**	**Protein description**
General stress-related proteins	Cold shock protein	*cspA*	Met	Up	1	Cordwell et al., 2002	Cold shock tolerance
	Dps family protein	*dps*	Van	Down	1	Ramos et al., 2015	Protection of DNA from damage
	Two-component regulator protein	*vanR*	Van	Up	1	Ramos et al., 2015	The VanS/VanR two-component system in response to extracellular glycopeptide antibiotic
	Lactoylglutathione lyase	*gloA*	Van	Up	1	Wang et al., 2010	Methylglyoxal degradation
	Cell stress stimulon response regulator	*vraR*	Van	Up	2	Scherl et al., 2006; Drummelsmith et al., 2007	The two-component regulatory system VraS/VraR involved in the control of the cell wall peptidoglycan biosynthesis
	HTH-type transcriptional regulator	*sarS*	Van	Down	1	Drummelsmith et al., 2007	Transcriptional regulator that controls expression of some virulence factors in a cell density-dependent manner
	Accessory gene regulator A	*agrA*	Van	Down	2	Scherl et al., 2006; Drummelsmith et al., 2007	The regulation of virulence proteins
	Signal transduction protein TRAP	*traP*	Van	Up	1	Scherl et al., 2006	A major regulator of staphylococcal pathogenesis
	Thioredoxin reductase	*trxB*	Van	Down	1	Drummelsmith et al., 2007	Thioredoxin pathway
	Competence protein	*cglA*	Lin	Down	1	Feng et al., 2011	Competence regulation
	Competence protein	*cglB*	Lin	Down	1	Feng et al., 2011	Competence regulation
	Phosphate transporter	*phoU*	Lin	Down	1	Feng et al., 2011	Phosphate starvation
	Conserved membrane protein; phage-shock protein A homolog (three-component regulatory system)	*liaIH*	Dap	Up	1	Wecke et al., 2009	Regulation of membrane permeability
	Undecaprenyl pyrophosphate phosphatase	*bcrC*	Dap	Up	1	Wecke et al., 2009	Bacitracin resistance
	Superoxide dismutase	*sodM*	Met	Up	2	Cordwell et al., 2002; Enany et al., 2014	Resistance to oxidative stress
			Van	Down	1	Drummelsmith et al., 2007	
	Competence damage-inducible protein A	*cinA*	Van	Up	1	Pieper et al., 2006	Competence regulation
			Lin	Down	1	Feng et al., 2011	
	Two-component sensor histidine kinase	*vraS*	Van	Up	1	Scherl et al., 2006	The two-component regulatory system VraS/VraR involved in the control of the cell wall peptidoglycan biosynthesis
			Dap	Up	1	Fischer et al., 2011	
	Staphylococcus accessory regulator A	*sarA*	Van	Up	1	Drummelsmith et al., 2007	Regulation of the virulence factors
			Dap	Up	1	Fischer et al., 2011	
	GTP pyrophosphokinase	*relA*	Van	Down	1	Drummelsmith et al., 2007	Stringent response
			Dap	Up	2	Wecke et al., 2009; Fischer et al., 2011	
	Choline dehydrogenase	*betA*	Van	Up	1	Scherl et al., 2006	Glycine betaine biosynthesis
			Dap	Down	1	Fischer et al., 2011	
	GTP-sensing transcriptional pleiotropic repressor	*codY*	Lin	Up	1	Feng et al., 2011	Transcription regulation in response to the GTP level
			Dap	Up	1	Fischer et al., 2011	
	Alkaline shock protein 23	*asp23*	Met	Up	2	Cordwell et al., 2002; Enany et al., 2014	Alkaline pH tolerance
			Van	Down	1	Hessling et al., 2013	
			Dap	Up	1	Fischer et al., 2011	
	Catalase	*katA*	Met	Up	1	Cordwell et al., 2002	Resistance to oxidative stress
			Van	Up	1	Scherl et al., 2006	
			Dap	Up	1	Fischer et al., 2011	
	Superoxide dismutase	*sodA*	Met	Up	2	Cordwell et al., 2002; Enany et al., 2014	Resistance to oxidative stress
			Van	Up	1	Wang et al., 2010	
			Lin	Up	1	Feng et al., 2011	
	Cold shock protein	*cspB*	Met	Up	1	Cordwell et al., 2002	Cold shock tolerance
			Van	Down	1	Drummelsmith et al., 2007	
			Dap	Up	1	Fischer et al., 2011	
	Cold shock protein	*cspC*	Met	Up	1	Cordwell et al., 2002	Cold shock tolerance
			Van	Down	1	Drummelsmith et al., 2007	
			Dap	Up	1	Fischer et al., 2011	
	Alkyl hydroperoxide reductase subunit C	*ahpC*	Met	Up	1	Enany et al., 2014	Resistance to oxidative stress
			Van	Up	2	Scherl et al., 2006; Chen et al., 2013	
			Dap	Up	1	Fischer et al., 2011	
Virulence-related proteins	Secreted virulence factor	*esxA*	Van	Down	1	Drummelsmith et al., 2007	Pathogenesis
	Extracellular ECM and plasma binding protein	*ssp*	Van	Up	1	Scherl et al., 2006	Pathogenesis
	Cell surface-associated protein	*sdrE*	Van	Down	1	Hessling et al., 2013	Pathogenesis
	Clumping factor A	*clfA*	Van	Down	1	Hessling et al., 2013	Pathogenesis
	Secretory extracellular matrix and plasma binding protein	*empbp*	Van	Down	1	Hessling et al., 2013	Pathogenesis
	Enterotoxin type I	*sei*	Van	Down	1	Hessling et al., 2013	Pathogenesis
	Cysteine protease precursor	*sspB1*	Van	Down	1	Hessling et al., 2013	Pathogenesis
	Leukotoxin	*lukD*	Van	Down	1	Hessling et al., 2013	Pathogenesis
	Leukotoxin	*lukE*	Van	Down	1	Hessling et al., 2013	Pathogenesis
	Phospholipase C	*hlb*	Van	Down	1	Hessling et al., 2013	Pathogenesis
	HysA	*hysA*	Van	Down	1	Hessling et al., 2013	Pathogenesis
	γ-hemolysin, component C	*hlgC*	Van	Down	1	Hessling et al., 2013	Pathogenesis
	Lipase	*geh*	Van	Down	1	Hessling et al., 2013	Pathogenesis
	Accessory protein Z	*sarZ*	Van	Down	1	Hessling et al., 2013	Pathogenesis
	α-hemolysin	*SAV1163*	Lin	Down	1	Bernardo et al., 2004	Pathogenesis
	Respiratory response protein	*srrA*	Met	Up	1	Cordwell et al., 2002	Pathogenesis
			Van	Down	1	Scherl et al., 2006	
	Fibrinogen-binding protein	*efb*	Met	Up	1	Enany et al., 2014	Pathogenesis
			Van	Down	1	Hessling et al., 2013	
	Immunoglobulin G binding protein A	*spa*	Van	Down	2	Pieper et al., 2006; Drummelsmith et al., 2007	Pathogenesis
			Lin	Down	1	Bernardo et al., 2004	
	Bifunctional autolysin	*atl*	Van	Up	1	Drummelsmith et al., 2007	Pathogenesis; Cell wall biogenesis/degradation
			Lin	Down	1	Bernardo et al., 2004	
			Dap	Up	1	Fischer et al., 2011	
	Immunodominant antigen A	*isaA*	Met	Up	1	Cordwell et al., 2002	Pathogenesis; Cell wall biogenesis/degradation
			Van	Up	3	Scherl et al., 2006; Drummelsmith et al., 2007; Chen et al., 2013	
			Lin	Down	1	Bernardo et al., 2004	
			Dap	Up	1	Fischer et al., 2011	

Hassling et al. analyzed proteomic profiles of vancomycin-susceptible *S. aureus* strain COL under the sublethal vancomycin exposure (4.5 μg/ml) (Hessling et al., 2013). They found the specific increase of proteins involved in the synthesis of lysine which are essential for the synthesis of the peptidoglycan precursor pentapeptide; aspartate kinase (LysC), aspartate semialdehyde dehydrogenase (Asd), dihydrodipicolinate synthase (DapA), 4-hydroxy-tetrahydrodipicolinate reductase (DapB), diaminopimelate decarboxylase (LysA), and tetrahydrodipicolinate acetyltransferase (DapD). An increase of lysine synthesis proteins can lead to an overall increase of peptidoglycan synthesis. Induction of genes involved in lysine synthesis under cell wall stress conditions have been documented before by two transcriptome studies (Kuroda et al., 2003; Sobral et al., 2007). Consistent with the previous report (Scherl et al., 2006), this report also showed that several proteins regulated by the two-component system VraSR increased in amount after vancomycin addition (Hessling et al., 2013). Additionally, they identified two important regulators (the alternative sigma factor σ^B^ and the two-component system SaeRS regulating numerous virulence genes) that play a role in vancomycin stress response. The cluster of proteins under positive σ^B^ control mainly increased, whereas negatively regulated proteins primarily decreased in amount after vancomycin addition (Hessling et al., 2013). The induction of σ^B^ regulon by vancomycin has been found in another report (Chen et al., 2013). Increase of the σ^B^ activity has also been observed in strains resistant to teicoplanin (Bischoff and Berger-Bächi, 2001) or methicillin (Cordwell et al., 2002). Hassling et al. also found decreased expression levels of most proteins with a virulence related function (Hessling et al., 2013). However, because the great majority of virulence genes in previous transcriptome studies under cell wall stress in *S. aureus* have been shown to be up-regulated (Kuroda et al., 2003; Utaida et al., 2003; Sobral et al., 2007), the role of virulence genes in antibiotic resistance needs to be determined.

Lastly, Wang et al. and Ramos et al. performed proteomic analysis of vancomycin-resistant *E. faecalis* strains (V583, V306, and SU18) under 64 μg/ml vancomycin treatment (Wang et al., 2010; Ramos et al., 2015). Vancomycin induced expression of vancomycin resistance-related proteins such as VanA, VanX, D-Ala-D-Ala dipeptidase (DdpX), VanR, and VanB (Wang et al., 2010; Ramos et al., 2015). Distinctively, Wang et al. found that six proteins (Pgm, Ldh, Gap-2, RpsB, EF2076, and sex pheromone cAD1 precursor lipoprotein) exhibited clear post-translational modifications and vancomycin induced phosphorylation of Ser/Thr in Ldh, Gap-2, and sex pheromone cAD1 precursor lipoprotein (EF3256) (Wang et al., 2010). Ramos et al. showed that metabolism-related proteins, such as TipA, GMP synthase (GuaA), and glyceraldehyde-3-phosphate dehydrogenase (GapB), were down-regulated under vancomycin treatment (Ramos et al., 2015).

### Linezolid

There was one study exploring comparative proteomic profiles in linezolid-susceptible *S. pneumonia* strains and linezolid-resistant *S. pneumonia* strains, and one study analyzing global proteomes of a linezolid- susceptible *S. aureus* under linezolid stresses (Bernardo et al., 2004; Feng et al., 2011). Through the comparison between linezolid-susceptible *S. pneumonia* strains (1974 and R6) with linezolid MICs of 0.5–0.75 μg/ml and linezolid-resistant *S. pneumonia* strains (1974M2-LZD and R6M2-LZD) with MIC of 32 μg/ml, Feng et al. showed that the proteomic and transcriptomic approaches were poorly correlated with previously known resistance factors (23S rRNA, ribosomal proteins L3 and L4, RNA methyltransferase Cfr, and ABC transporter PatA and PatB), as modulated proteins rarely had significant concomitant changes at the expression level (Feng et al., 2011). They found increased expression of proteins involved in the metabolism and transport of carbohydrates in linezolid-resistant *S. pneumoniae* strains (Feng et al., 2011). Through inactivation of target genes in the linezolid-resistant strains (1974M2-LZD and R6M2-LZD), they identified two ABC transporter substrate-binding proteins (Spr0083 and Spr1527) and the catabolite control protein A (CcpA) as factors associated with resistance to linezolid (Feng et al., 2011). CcpA is known to function as the global regulator controlling the efficient utilization of sugars through carbon catabolite repression (CCR) in Gram-positive bacteria (Stülke and Hillen, 2000). Inactivation of the *ccpA* gene in *S. aureus* affected growth, glucose metabolism, and expression of virulence genes (Seidl et al., 2006). CcpA inactivation was also linked to the down-regulation of glycolytic genes in *Bacillus cereus* (van der Voort et al., 2008; Feng et al., 2011). Therefore, the increased level of CcpA may cause the increased expression of glycolytic enzymes in linezolid-resistant *S. pneumonia* strains. In *S. aureus*, the correlation between antibiotic resistance and CcpA has already been reported, as CcpA inactivation significantly reduced the oxacillin resistance levels in MRSA and the teicoplanin resistance level in a glycopeptide-intermediate-resistant *S. aureus* strain (Seidl et al., 2006). Table 3 shows the possibility that CcpA may also be involved in methicillin and vancomycin resistance. Together with CcpA, inactivation of two ABC transporters putatively involved in the sugar transport (Spr0083 and Spr1527) also reduced resistance to linezolid of *S. pneumonia* (Feng et al., 2011). Notably, *S. pneumoniae* is predicted to be highly dependent on external sugars to fulfill its energy requirements by substrate-level phosphorylation as it lacks functional electron transport chain and tricarboxylic acid cycle (Tettelin et al., 2001; Feng et al., 2011). This process eventually leads to the formation of lactate and acetate by the lactate dehydrogenase and lactate oxidase enzymes and these proteins were also found to be overexpressed in linezolid-resistant *S. pneumonia* strains (Tettelin et al., 2001; Feng et al., 2011). Therefore, these results imply increased energy requirements associated with resistance mechanism to linezolid in *S. pneumonia* (Feng et al., 2011). To sustain a fitness cost associated with resistance mechanisms such as the 23S rRNA mutations (Besier et al., 2008), *S. pneumonia* seems to select an increased metabolism of sugars as a secondary adaptation.

This study also showed that several genes involved in the biosynthesis of fatty acids, including enoyl-CoA hydratase (PhaB), 3-ketoacyl-ACP reductase (FabG), acetyl-CoA carboxylase biotin carboxyl carrier protein subunit (AccB), acetyl-CoA carboxylase subunit alpha (AccA), and hydroxymyristoyl-ACP dehydratase (FabZ), were down-regulated in linezolid-resistant strains (Feng et al., 2011). Whether this is directly related to linezolid resistance remains to be established, but it is intriguing that the cell wall inhibitor penicillin also causes a down-regulation of several genes of this pathway in *S. pneumoniae* (Rogers et al., 2007; Feng et al., 2011). Interestingly, expression levels of FabZ are changed in all cases of the four antibiotics (Table 6), even though its expression increased in methicillin-resistant strains and decreased in strains resistant to other antibiotics. Many numbers of ribosomal proteins were found to be overexpressed or down-regulated in linezolid-resistant strains, but whether this pattern is due to the mechanism of action of linezolid (which targets the ribosome) remains to be established. Although recent several lines of evidence indicate the presence of functional selective ribosomal subpopulations that exhibit variations in the RNA or the protein components and modulate the translational program in response to environmental changes (Byrgazov et al., 2013), it is difficult to obtain any information from variation patterns of ribosomal proteins in this study.

Bernardo et al. compared the change of proteomic profiles of a linezolid- susceptible *S. aureus* strain ATCC 29213 (MIC = 2.5 μg/ml) under linezolid stresses (12.5, 25, 50, and 90% of MIC) (Bernardo et al., 2004). They found that linezolid reduced in a dose-dependent manner the secretion of specific virulence factors, including bifunctional autolysin (Atl), immunoglobulin G binding protein A (Spa), and α-hemolysin (SAV1163), CHAP-domain amidase (SsaA), and immunodominant antigen A (IsaA). This result is similar to the proteomic result that analyzes protein profiles of *S. aureus* under the sublethal vancomycin exposure (Hessling et al., 2013).

### Daptomycin

There were one study examining comparative proteomic profiles in daptomycin-susceptible and daptomycin-resistant *S. aureus* strains, and one study analyzing global proteomes of daptomycin-susceptible *B. subtilis* under daptomycin stress (Wecke et al., 2009; Fischer et al., 2011). Unlike other three antibiotics (methicillin, vancomycin, and linezolid), specific genetic determinant of the daptomycin-resistant strain was not determined. Probable daptomycin resistance-related proteins (MprF, YycG, RpoB, and RpoC) identified in previous reports (Jones et al., 2008; Baltz, 2009) were not identified in comparative proteomic analyses (Tables 2–8). In 2011, Fisher et al. compared proteomic profiles in the daptomycin-susceptible *S. aureus* strain 616 with a daptomycin MIC of 0.5 μg/ml and the daptomycin-resistant *S. aureus* strain 701 with MIC of 2 μg/ml (Fischer et al., 2011). Comparative proteomics and transcriptomic approach revealed a differential abundance of proteins in various functional categories, including cell wall-associated targets and biofilm formation proteins (Fischer et al., 2011). Phenotypically, daptomycin-susceptible strains, and daptomycin-resistant strains showed major differences in their ability to develop bacterial biofilms in the presence of the antibacterial lipid, oleic acid (Fischer et al., 2011). Transcriptomic approach showed different expressions of some important genes, such as the key genes (*yycFGHI*) affecting cell membrane lipid homeostasis, cell wall metabolism and biofilm formation, and two-component regulation system genes (*agr, saeRS*, and *vraRS*) involved in pathogenesis of methicillin-resistant strains (Fischer et al., 2011). However, through proteomic research, only several proteins, including Asp23, 3-oxoacyl-ACP synthase II (FabF), GTP-sensing transcriptional pleiotropic repressor (CodY), and PurH, was identified as proteins involved in daptomycin resistance.

**Table 8:** **Differentially expressed proteins identified by the quantitative proteomic approach: proteins of unknown function**.

**Biological process**	**Protein name**	**Gene**	**Antibiotics**	**Regulation**	**Frequency of difference**	**References**	**Protein description**
General function prediction only	Metal-dependent hydrolase	*SA1529*	Met	Down	1	Cordwell et al., 2002	Protein degradation
	Aldehyde dehydrogenase	*SAV2122*	Met	Up	1	Enany et al., 2014	Energy metabolism
	Putative transaldolase	*tal*	Van	Down	1	Drummelsmith et al., 2007	Energy metabolism
	Putative transcription factor	*SA2296*	Van	Up	1	Scherl et al., 2006	Gene expression
	Penicillin binding methicillin resistant-related protein	*fmtA*	Van	Up	1	Scherl et al., 2006	Peptidoglycan biosynthesis
	Putative cell wall transglycosylase	*sceD*	Van	Up	1	Pieper et al., 2006	Peptidoglycan degradation
			Lin	Up	1	Bernardo et al., 2004	
	ABC protein/substrate binding protein subunit—Sugar transport	*spr0083*	Lin	Up	1	Feng et al., 2011	Carbohydrate transport
	ABC protein/substrate binding protein subunit—sugar transport	*spr1527*	Lin	Up	1	Feng et al., 2011	Carbohydrate transport
	Maltose/maltodextrin-binding protein	*SA0207*	Dap	Down	1	Fischer et al., 2011	Carbohydrate transport
Function unknown	Unknown	*SA1238*	Met	Up	1	Cordwell et al., 2002	Unknown
	Unknown	*SA1051*	Met	Up	1	Cordwell et al., 2002	Unknown
	Unknown	*SA0940*	Met	Up	1	Cordwell et al., 2002	Unknown
	Unknown	*SA1868*	Met	Up	1	Cordwell et al., 2002	Unknown
	Unknown	*SA1813*	Met	Up	1	Cordwell et al., 2002	Unknown
	Unknown	*SA2302*	Met	Down	1	Cordwell et al., 2002	Unknown
	Unknown	*SA0759*	Met	Down	1	Cordwell et al., 2002	Unknown
	Unknown	*SA1812*	Met	Up	1	Cordwell et al., 2002	Unknown
	Unknown	*SA0587*	Met	Up	1	Cordwell et al., 2002	Unknown
	Unknown	*SA0772*	Met	Up	1	Cordwell et al., 2002	Unknown
	Unknown	*SA0587*	Met	Up	1	Cordwell et al., 2002	Unknown
	Unknown	*SA1455*	Met	Up	1	Cordwell et al., 2002	Unknown
	Unknown	*SA0919*	Met	Down	1	Cordwell et al., 2002	Unknown
	Unknown	*SA1709*	Met	Down	1	Cordwell et al., 2002	Unknown
	Unknown	*SA0022*	Van	Down	1	Scherl et al., 2006	Unknown
	Unknown	*SA2113*	Van	Up	1	Scherl et al., 2006	Unknown
	Unknown	*vraX*	Van	Up	1	Scherl et al., 2006	Unknown
	Unknown	*SA0423*	Lin	Down	1	Bernardo et al., 2004	Unknown
	Unknown	*SAV0719*	Lin	Down	1	Bernardo et al., 2004	Unknown
	Unknown	*spr1987*	Lin	Down	1	Feng et al., 2011	Unknown
	Unknown	*spr0033*	Lin	Up	1	Feng et al., 2011	Unknown
	Unknown	*spr0125*	Lin	Down	1	Feng et al., 2011	Unknown
	Unknown	*spr0895*	Lin	Up	1	Feng et al., 2011	Unknown
	Unknown	*spr0618*	Lin	Up	1	Feng et al., 2011	Unknown
	Unknown	*spr0997*	Lin	Down	1	Feng et al., 2011	Unknown
	Unknown	*spr1332*	Lin	Up	1	Feng et al., 2011	Unknown
	Unknown	*spr1693*	Lin	Up	1	Feng et al., 2011	Unknown
	Unknown	*spr1726*	Lin	Up	1	Feng et al., 2011	Unknown
	Unknown	*spr1758*	Lin	Down	1	Feng et al., 2011	Unknown
	Unknown	*spr2029*	Lin	Down	1	Feng et al., 2011	Unknown
	Unknown	*spr0174*	Lin	Up	1	Feng et al., 2011	Unknown
	Unknown	*spr0370*	Lin	Up	1	Feng et al., 2011	Unknown
	Unknown	*SA0269*	Dap	Down	1	Fischer et al., 2011	Unknown
	Unknown	*SA0591*	Van	Up	1	Scherl et al., 2006	Unknown
	Unknown	*SA1528*	Met	Down	1	Cordwell et al., 2002	Unknown
			Dap	Down	1	Fischer et al., 2011	

Wecke et al. searched proteins induced by daptomycin, through the proteomic approach of a daptomycin-susceptible *B. subtilis* strain W168 under daptomycin treatment of sublethal amount (1 μg/ml) (Wecke et al., 2009). They identified LiaI and LiaH proteins exclusively and strongly induced (429-fold) by daptomycin. This result is in good agreement with data analyzing genes induced by daptomycin through transcriptome profiling (Muthaiyan et al., 2008). LiaH is a conserved membrane protein similar to a phage shock protein A (PspA) of *E. coli*, and its expression is regulated by the cell envelope stress-sensing two-component system LiaRS (Jordan et al., 2006; Hachmann et al., 2009; Wecke et al., 2009). Inactivation of *liaH* leads to 3-fold increased susceptibility to daptomycin and this susceptibility was further exacerbated in cells additionally lacking the paralogous gene *pspA* (Hachmann et al., 2009). In *E. coli*, the *pspA* gene is induced upon phage infection, osmotic shock, exposure to ethanol, or temperature increase, and functions to help cells manage the impacts of agents impairing cell membrane function (Joly et al., 2010). A recent report showed that deletion of the response regulator LiaR regulating expression of *liaIH* in daptomycin-resistant *E. faecalis* reversed resistance to daptomycin, and resulted in hypersusceptibility to daptomycin (Reyes et al., 2015). Therefore, these results indicate that LiaR is a master regulator protecting cell membrane to diverse antimicrobial agents, through regulating expression of various genes such as the *liaH* gene (Reyes et al., 2015).

## Conclusion

Although specific genetic determinants of resistance mechanisms to methicillin, vancomycin, and linezolid were identified through non-proteomic approaches (e.g., *van* genes in vancomycin resistance) (Table 1), recent comparative proteomic methods provide new opportunities to understand the antibiotic resistance mechanism. In particular, in the case of recently used antibiotics such as daptomycin, specific genetic determinant(s) of antibiotic resistance was not fully determined through non-proteomic approaches. Therefore, quantitative proteomic methods can be a good tool to find an important protein involved in daptomycin resistance. Actually, a proteomic research identified LiaH as a highly induced protein by daptomycin treatment (Muthaiyan et al., 2008) and a subsequent report showed that the expression level of this protein is important to daptomycin-resistant phenotype (Reyes et al., 2015). These results show that quantitative proteomic analysis can be used as an effective tool to find novel resistance mechanisms.

Interestingly, comparative proteomic approaches in methicillin, linezolid, and daptomycin, except for vancomycin, were poorly correlated with known resistance-related factors found by non-proteomic approaches (Table 2). This result may be caused by a lack of comparative proteomic studies in three antibiotics, or imply the existence of novel resistance mechanisms different from previously known resistance mechanisms found by non-proteomic approaches. Through summarizing recent data of comparative proteomic researches of four clinically important antibiotics, we can find proteins of which expression levels are changed only in the resistance mechanism to specific antibiotic, such as LiaH in daptomycin resistance and PrsA in vancomycin resistance. It is necessary to determine whether these proteins affect antibiotic resistance through regulating previously known resistance-related determinants or by a novel mechanism. Another interesting result is that many proteins identified by comparative proteomic analyses seem to be simultaneously involved in resistance mechanism to two or more antibiotics (Tables 2–8). These proteins include cold shock proteins (CspABC), sporulation protein G (SpoVG), alkyl hydroperoxide reductase subunit C (AhpC), L-lactate dehydrogenase (LdhA), triacylglycerol lipase precursor (LipA), superoxide dismutase (SodA), catalase (KatA), elongation factor G (FusA), CHAP-domain amidase (SsaA), two component system (VraSR), penicillin binding methicillin resistant-related protein (FmtA), adenylosuccinate lyase (PurB), glucose-6-phosphate isomerase (Pgi), catabolite control protein A (CcpA), putative cell wall transglycosylase (SceD), immunodominant antigen A (IsaA), bifunctional autolysin (Atl), the σ^B^ regulon, and hydroxymyristoyl-ACP dehydratase (FabZ). These proteins can be divided into two groups, proteins involved in bacterial envelope regulation and proteins compensating for a fitness cost of antibiotic resistance. Proteins such as LipA, VraSR, FmtA, SsaA, SceD, IsaA, Atl, and FabZ, are directly or indirectly involved in envelope regulation. In order to modify or thicken the bacterial cell wall for antibiotic resistance, cells require abundant energy, and proteins involved in stress adaptation are necessary to neutralize various damages by antibiotic. To sustain these fitness costs associated with resistance mechanisms, proteins involved in energy metabolism (LdhA, FusA, Pgi, PurB, and CcpA) and stress-related proteins (CspABC, SpoVG, AhpC, SodA, KatA, and the σ^B^ regulon) seem to be identified in resistance mechanisms to several antibiotics. Therefore, these proteomic results confirm that antibiotic resistance requires a fitness cost.

Detailed studies on the mechanism by which these proteins affect antibiotic resistance are required. In particular, because these proteins can act as the global factor affecting resistance mechanisms to most antibiotics, it is necessary to examine whether they affect resistance mechanism of other antibiotics having different action modes. These studies will provide important clues for understanding and managing antibiotic resistance.

### Conflict of interest statement

The authors declare that the research was conducted in the absence of any commercial or financial relationships that could be construed as a potential conflict of interest.
